# Comparison of torch with electric arc casting in the lost wax technique for the cast dental stud protocol

**DOI:** 10.1371/journal.pone.0321724

**Published:** 2025-04-29

**Authors:** Víctor Hugo Gonzalo Quispe Mamani, César Alberto Luna Castillo, Dina Miryan Alanoca Sejje, Luis Alexander Orrego-Ferreyros

**Affiliations:** Universidad César Vallejo, Facultad de Ciencias de la Salud, Escuela Profesional de Estomatología, Piura, Perú; UNICAMP, University of Campinas, BRAZIL

## Abstract

The aim of this study was to compare the compressive strength, microstructural characteristics, and cost-effectiveness of cast dental posts fabricated using two techniques: torch casting (TC) and electric arc casting (EAC), both integral to the lost wax method. Employing an applied research approach with an experimental design, the study analyzed 40 non-precious gold (NPG) alloy cast posts, divided equally into two groups of 20 for each technique. The selection process was non-probabilistic and based on convenience, with specific inclusion and exclusion criteria to ensure precision and relevance. The results reveal a significant advantage for the EAC technique. In Essay 1, EAC posts exhibited a mean compressive strength of 206.102 MPa, compared to 157.207 MPa for TC posts. Similarly, in Essay 2, EAC posts showed a mean strength of 172.625 MPa versus 136.303 MPa for TC posts. These differences were statistically significant (p < 0.05), with EAC posts also displaying smaller failure diameters and areas, suggesting better load distribution. Morphological and microstructural analysis using scanning electron microscopy (SEM) revealed a porous surface with irregular topography in both techniques. However, EAC samples displayed crystalline growths within the copper matrix, indicating a non-homogeneous stoichiometry, while TC samples showed aluminum-enriched zones, suggesting a non-uniform elemental distribution. Chemical composition analysis via energy-dispersive spectroscopy (EDS) identified copper (Cu) as the predominant element in both samples, with trace elements such as aluminum (Al), nickel (Ni), and iron (Fe) also present. X-ray diffraction (XRD) analysis further revealed distinct crystalline phases, with EAC samples exhibiting a higher proportion of Cu₃Zn and gamma-Fe phases compared to TC samples. A cost analysis using Python 3.13 and Monte Carlo simulation with 1,000 iterations revealed that EAC is more expensive, with a total cost per unit of 2.181compared to 1.467 for TC, primarily due to higher operational costs. The Mann-Whitney U test confirmed significant differences in cost distributions (p < 0.001), indicating that EAC has higher and more variable costs. In conclusion, the study demonstrates that EAC produces dental models with significantly higher compressive strength and a more refined microstructure compared to TC, enhancing restoration durability. However, its higher operational costs must be considered. These findings provide valuable information for dental professionals, particularly in low- to middle-income countries, and suggest that future research should explore additional properties such as corrosion resistance and biocompatibility to further validate the clinical applicability of these materials.

## 1. Introduction

Dentistry, as a constantly evolving discipline, is characterized by its focus on continuous improvement and the adoption of advanced methods and technologies for the diagnosis and treatment of dental diseases [1,2]. In this context, metals play a key role in dentistry, as they are used in a wide variety of applications ranging from the fabrication of prosthetic devices and orthodontic bands to the creation of temporary and permanent dental crowns, as well as direct dental restorations [3–5]. One of the key methods for obtaining wax-based metal models in dentistry is the lost-wax casting process [6–9]. This method has become an important pillar in metallurgy and is widely used in the creation of high-quality dental components. At present, two main approaches to casting in dentistry stand out: electric arc casting and torch casting [7,10–12]. Both methods have proven their effectiveness and versatility in the creation of dental metal frameworks, and the choice between them often depends on specific factors related to the clinical case and the dentist’s preferences. The electric arc melting process represents an advanced technique in which an electric arc is used to melt the metal. What distinguishes this method is its high metal melting speed, which results in remarkably fast melting. In addition, it excels in uniform and precise heat distribution during the process. This uniform distribution is essential, as it leads to a more homogeneous melt in terms of structure and quality [13–16]. On the other hand, torch melting is another fundamental approach in obtaining metal parts in dentistry. In this process, a torch is used to melt the metal. The outstanding feature of torch melting is its ability to maintain a very precise temperature, resulting in less variability in the casting process. In addition, this method tends to generate a lower amount of porosity in the melt, which is crucial for high quality results [17,18]. The choice between electric arc casting and torch casting in dentistry poses an interesting challenge, as both methods have their own advantages and disadvantages [7,16]. On the one hand, electric arc casting is noted for its speed in the metal melting process and uniform heat distribution, resulting in a homogeneous casting. However, it may have certain limitations or disadvantages that should be evaluated in comparison with other methods. On the other hand, torch melting offers precision in temperature control and a lower propensity for porosity formation in the casting, which is critical for obtaining high quality metal parts. However, it may also have some limitations or challenges that need to be considered. Since there is still no clear consensus in the dental community as to which of these methods is the most suitable for the lost wax technique in the protocol, it becomes essential to carry out a detailed comparison between the two. This involves evaluating their mechanical properties and performing compressive strength tests, among other relevant analyses. The evaluation of mechanical properties and compressive strength becomes a crucial tool to determine which of these methods is more suitable for the fabrication of a cast dental model. These studies provide quantitative data and empirical results that can shed light on which of the two methods is the most appropriate for a specific context. The aim of this study was to compare the compressive strength, microstructural characteristics, and cost-effectiveness as part of the material science evaluation protocol for cast dental posts fabricated using two techniques: torch casting (TC) and electric arc casting (EAC), both integral to the lost wax method.

## 2. Materials and methods

The research is applied, with an experimental design. The sample consisted of 40 non-precious gold (NPG) colour alloy cast posts (20 posts fabricated using the Torch Casting technique and 20 using the Electric Arc Casting technique). The sampling was probabilistic, simple random. The study established specific inclusion and exclusion criteria to ensure the precision and relevance of its findings, focusing on the characteristics of cast posts used in natural teeth, specifically the upper or lower second premolars. For inclusion, several key criteria were defined. Firstly, the posts were casted from the upper or lower unique conduct second premolars. The post’s length was another crucial factor; it needed to be long enough to ensure both good retention and support for the tooth. This length was standardized at 16 mm, comprising 4 mm for the stump and 12 mm for the spike. The diameter of the post was also a significant consideration. It had to be large enough to provide adequate retention without compromising the tooth’s structural integrity. To this end, the diameter at the apical edge was set at 1.5 mm, and at the coronal edge at 3.5 mm. Additionally, the shape of the post was important; it needed to fit snugly into the root canal of the tooth. The material specified for the post was NPG, chosen for its frequent use as reported by many dentists. On the other hand, the exclusion criteria were equally stringent to maintain the study’s integrity. Cast posts with bubbles were excluded, as these could signify flaws in the casting process, potentially affecting the strength and reliability of the posts. Similarly, posts with any altered morphology were not considered for the study, ensuring that only standard, uniformly shaped posts were evaluated. This careful approach to selecting the characteristics of the cast posts aimed to produce results that were applicable to real-world dental practices. Five posts were fabricated in a testing phase for each technique to fine-tune the characteristics that the sample posts should have. These were not considered in the analysis.

### 2.1. Instruments and procedures

#### 2.1.1. Laboratory process.

For the pre-laboratory process of endodontic preparation and direct impression with Duralay® for the elaboration of the post or pin, the procedure described by Gómez was followed [19].

The impression was sent to the dental laboratory where the customized dental pins were fabricated to the required shape and size. In the manufacturing process, acrylic dies were used, with a total of 50 copies made in full-fired acrylic (Duralay®) from the model provided. Additionally, sprues were implemented with a T-shaped main channel, which had a diameter of 4 mm and a minimum distance of 10 mm from the casting funnel to the bar. The bar itself had a diameter of 4 mm, and the main channel had a protrusion on the bar between two joints. The splice leading to the casting object had a diameter of 3 mm and a length of 3 mm. Each single post was located at the highest point of the cast piece.

To avoid the inclusion of gases during the casting process, a system of cylinders with specific shapes and cones for casting was used. The base of this system was kept completely clean. During the mounting of the casting in the casting cone, special attention was paid to the connection between the casting and the cone, ensuring that it was precise and clean to guarantee the optimum flow of the cast metal. The object to be cast was mounted horizontally, centred inside a Plexiglas ring, with a minimum distance of 6 mm from the edge. A transparent cylinder ring was inserted, ensuring that the top edge of the cast object was 6 mm below the edge of the cylinder ring. This ensured that the sprue was 15–20 mm long, and the cylinder was filled to the top evenly.

The coating process was essential to obtain a stable cylinder and accurate casting. For this purpose, the coating processing instructions (Rematitan Plus) were strictly followed. The consumption of Rematitan Plus investment was calculated based on the 62 x 250 g size. Mixing of the coating was carried out only with the corresponding mixing liquid, which was kept at a suitable temperature. The mixing ratio was 250 g of powder per 40 ml of mixing liquid, and mixing was carried out for 60 seconds. Once prepared, the cylinder was filled to the brim and removed from the vibrator. The hardening required approximately 40 minutes, and afterward, it was dry trimmed from the opposite side to the form-cone to favor the permeability of the coating in the appropriate direction.

The expansion control of the Rematitan Plus investment was carried out by dilution of the mixing liquid, following the specific recommendations for different types of work, such as crowns and bridges or telescopic and conical crowns. The furnace was preheated to the required final temperature of 1000°C using a specific program, with a holding time in different stages and slow cooling down to 430°C, the casting temperature. It was important to avoid sudden temperature changes and to maintain the final temperature at 430°C for a maximum of 120 minutes. Only waxes or plastics that did not leave residues were used.

The required preheating furnace had a heating chamber on three or four sides, good insulation, and programmable control. The required final temperature was 1000°C, and air circulation was recommended. The furnace capacity was not completely filled to avoid cracks in the cylinders due to poor insulation or rapid heating.

The procedures were carried out in a private dental laboratory.

#### 2.1.2. Casting process.

**a. Torch Casting:** The oxyacetylene torch casting technique was a widely used procedure for melting metals by creating a flame generated by combining oxygen and acetylene. To carry out this process effectively, several key steps were followed.

First, equipment and materials were prepared. Before starting, it was essential to ensure that all welding equipment and supplies were in optimal condition and properly connected. Additionally, it was important to have sufficient oxygen and acetylene to carry out the melting process. A welding torch with the proper nozzle for the task at hand was selected.

Next, the metal was prepared. The type of metal to be cast was chosen according to the specific needs of the project. The metal was cleaned thoroughly to remove any traces of rust, dirt, or contaminants that might have affected the quality of the casting.

The torch ignition followed. The oxygen and acetylene valves on the cylinder regulators were gradually opened. The torch was ignited using an igniter designed for welding torches, and the gas flow was adjusted according to the manufacturer’s recommendations.

Once the torch was lit, the oxygen and acetylene valves were adjusted to obtain a neutral or slightly oxidizing flame. A suitable flame was blue in colour and well-defined, without the presence of yellow plumes or dark shadows.

The torch flame was then directed towards the metal to be melted, maintaining an appropriate distance to ensure even heat distribution. The torch was moved constantly to prevent the metal from overheating or burning.

When the metal reached the proper melting temperature, pressure was applied, or additional tools were used to shape or pour the metal into the desired mold or container.

After melting, the molten metal was allowed to cool completely in the mold or vessel, following the recommended cooling guidelines for the specific metal. After cooling, any necessary finishing, or adjustments to the molten metal, such as cleaning, polishing, or removal of imperfections, were performed.

Throughout the casting process, rigorous safety measures were followed to protect both the operator and the environment. These measures included wearing proper personal protective equipment, working in well-ventilated areas, handling molten materials with care, preventing fires, and knowing the safe handling of combustible gases. Additionally, it was essential to be trained and familiar with safety protocols specific to the workplace.

Upon completing this process, we initially produced 10 posts. The process was then repeated to generate an additional 10 posts.

**b. Electric Arc Casting**: For the electric arc casting process, an electric arc was created using a conventional digital inverter welder and electrodes, which consisted of Zinc (Zn) rods that acted as anode and cathode. When they came into contact, they generated an electric arc ideal for the melting process.

As for the ceramic crucible, it was used for non-precious alloys, with a maximum weight of 54 g, and for precious alloys with high gold content, with a maximum weight of 95 g. It was important to note that alloys with low precious metal content had a lower specific gravity, which reduced the maximum amount that could be melted compared to alloys with high gold content. A separate ceramic crucible had to be used for each type of alloy, and the crucible electrode was intended for only one type of alloy. Depending on the type of alloy used, the ceramic crucible could be reused up to 40 times and should not be cooled with water after casting to avoid possible breakage.

The melting electrode, located in the upper casting chamber, had to always keep its tips sharp, and its position remained constant. The distance between the two electrodes in alloy castings was 15 mm, with small variations of approximately 1 mm that did not affect the casting result.

The power of the melting and casting machine was adjustable by means of a rotary potentiometer and an inverter, which allowed the power to vary from 5% to 100% of the maximum power. The power regulation depended on both the melting temperature of the alloy and the amount of metal to be melted. To avoid the risk of cylinder cooling, the melting time should not have exceeded 40–50 seconds, and an automatic safety mechanism tipped the crucible after a maximum of 90 seconds. The suggestions for adjusting the power according to the quantity to be melted were indicative and could have varied according to the melting temperature and the state of the melting electrode.

During the casting process, it was visually monitored through a darkened window in the protective helmet. Caution was exercised at powers above 50% to avoid eye damage due to glare.

It was essential to pour the metal immediately after it was melted evenly, without delay, to avoid small blocks of metal remaining partially unmelted. The metal was stacked so that there was good contact with the crucible electrode and avoided accumulations on the back side of the crucible, where visibility was limited due to the electric arc.

After each melt, spatter residues were cleaned out, paying particular attention to any debris that may have remained in the funnel between the two chambers. In addition, the casting chamber window was cleaned regularly to observe the casting process clearly, and both chambers were cleaned regularly.

Upon completing this process, we initially produced 10 posts. The process was then repeated to generate an additional 10 posts.

#### 2.1.3. Physical measurements.

The measurements were made in a private physical materials analysis laboratory Congeomat.

We tested the compressive strength of the posts. Before the compression test, the weight of each sample was taken with a balance. The indication range of the balance was 200 g, with a scale division of 0.01 g, verification division 0.01 g, digital, OHAUS brand, model CS200, serial number 950037, from the United States. This instrument underwent a calibration procedure based on the Procedure for the Calibration of Non-Automatic Class III and IV Balances (PC-001) of the SNM-INDECOPI, 3rd edition January 2009 and the Peruvian Metrological Standard “Non-Automatic Weighing Instruments (NMP 003:2009)”. The maximum error observed in the verification series was 0.08 g at a load of 10g. The measuring instrument for the compression test was the CBR press with load cell, ARSOU brand, model PR401, series 41025, load cell type S, with digital indicator, of Peruvian origin. This instrument underwent a calibration procedure based on the ISO 7500–1 standard “Metallic materials - Verification of static uniaxial testing machines”. Two series of loads were applied to the Digital System using the same press. In each series the load readings were recorded. The maximum error observed in the verification series was 0.3% at a load of 500 kg. In addition, the diameter of the fault was measured. For this purpose, a 12-inch analog Vernier caliper from the brand Litz, made in the United States, was used as the instrument. This instrument underwent a calibration procedure using as reference the method described in PC-012: Calibration Procedure for caliper of the SNM-INDECOPI 5th Ed. The maximum error observed in the verification series was -0.09 mm for the reference block of 5.91 inches (150.00 mm). Using the diameter, we calculated the failure area.

#### 2.1.4. Morphological, microstructural, and chemical characterization.

For the morphological, microstructural, and chemical characterization, one dental post sample from each casting technique (electric arc and torch) was selected. These samples were analyzed to evaluate their surface morphology, internal microstructure, and chemical composition. The morphological analysis focused on aspects such as shape, size, and surface structure, while microstructural evaluation used microscopy techniques to examine the internal arrangement and structural features of the materials. Internal compositional analyses were conducted to determine the distribution and presence of chemical elements within the posts. Additionally, the chemical composition by phases of the cast models was established, providing a detailed view of the different phases present in the materials resulting from each casting technique.

Morphological and microstructural analysis was performed using a ThermoFisher PRISMA E scanning electron microscope (SEM) with a tungsten filament electron source, an acceleration voltage of 25 kV under high vacuum conditions, and secondary (ETD) and backscattered (CBS) electron detectors to obtain detailed micrographs of the post surfaces. Compositional analysis was conducted using energy-dispersive spectroscopy (EDS) to determine the elemental composition in terms of atomic and mass percentages. This analysis was performed on selected areas of polished surfaces, providing a detailed profile of the elements present. Chemical characterization was carried out using X-ray diffraction (XRD) with a Bruker D8-FOCUS diffractometer, employing a Cu tube (Kα1-Cu, λ = 1.5406 Å) and an angular range of 5° to 100° (2θ) with a step size of 0.02° and a step time of 1 second. The XRD analysis identified crystalline phases by comparing the obtained diffractograms with standardized databases, such as ICDD-2007.

Data analysis combined qualitative and quantitative methods. SEM micrographs were qualitatively analyzed to describe morphological and microstructural features, such as topography, elemental distribution, porosity, and surface irregularities. EDS data were quantitatively processed to calculate atomic and mass percentages, enabling precise evaluation of the chemical composition. XRD diffractograms were quantitatively analyzed using standardized databases (ICDD-2007) to identify and quantify crystalline phases, comparing results between electric arc and torch casting techniques.

The analysis was conducted in the X-ray Diffraction Laboratory of the Faculty of Physical Sciences at the National University of San Marcos.

### 2.2. Statistical analysis

The information collected was entered into a Microsoft 365 MS Excel spreadsheet and subsequently subjected to analysis using the STATA SE program in version 18.0.

Physical measurements analysis involved comparing the means of compressive strength (MPa) between Group No. 1 and Group No. 3, as well as between Group N° 2 and Group N° 4. We performed a normal distribution analysis, measures of central tendency and measures of dispersion of weight, failure diameter, failure area, maximum load (KN), maximum load (kg) and resistance obtained (Mpa). The Shapiro-Wilk test was used to evaluate normality. The inferential statistics used Student’s t test for independent samples with equal variances since the data had a normal distribution. We presented boxplots of compressive strength (MPa) for posts fabricated by electric arc melting and torch melting techniques in two experimental trials.

Morphological, microstructural, and chemical characterization was conducted, and relative frequencies were used to present the percentages of the elements and phases identified in the samples.

A cost analysis of casting techniques was conducted using Python 3.13 to evaluate the economic feasibility of torch casting and electric arc casting. To assess cost variability and uncertainty, a cost sensitivity analysis was performed using Monte Carlo simulation with 1,000 iterations. Adjusted probability distributions were applied, including Gamma for human resources and services, Log-Normal for materials, and PERT for depreciation and services. Additionally, the Mann-Whitney U test was used to compare the cost distributions between the two techniques. These methods were employed to model realistic cost uncertainty and variability, providing essential insights for decision-making in manufacturing and cost management.

### 2.3. Ethical considerations

The Research Ethics Committee (CIEI) of César Vallejo University reviewed and approved the execution of this research, as documented in the Research Ethics Committee Statement of the School of Stomatology No. 044–2023-/UCV/P.

## 3. Results

### 3.1. Physical measurements

**Fig 1:**
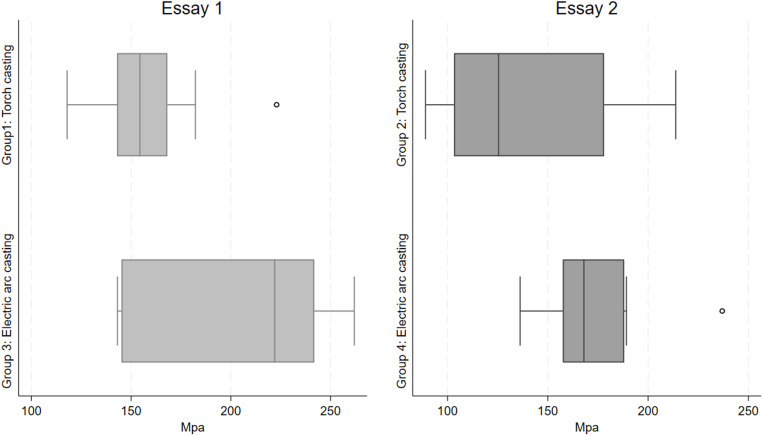
Boxplots of compressive strength (MPa) of posts fabricated by electric arc melting and torch melting techniques in two essays.

Table 1 and Fig 1 present a comparison of the physical and mechanical properties of posts fabricated using two different casting techniques: torch casting (TC) and electric arc casting (EAC). In Essay 1, it was observed that posts fabricated using torch casting (Group 1) had an average weight of 0.520 grams, a failure diameter of 0.155 cm, and a failure area of 0.019 cm². The maximum load supported by these posts was 0.290 KN (equivalent to 30.090 kg), with a mean resistance of 157.207 MPa. In comparison, posts fabricated using electric arc casting (Group 3) showed a slightly higher weight of 0.558 grams, a failure diameter of 0.150 cm, and a failure area of 0.017 cm². These posts supported a higher maximum load of 0.350 KN (36.770 kg) and had a mean resistance of 206.102 MPa. The average difference in compressive strength between the TC and EAC groups in this essay was -48.895 MPa, with a t-value of -2.698 and a p-value of 0.015, indicating a statistically significant difference.

**Table 1:** Comparison of physical and mechanical properties of posts fabricated by different casting techniques.

Casting technique used to obtain the cast post	Mean	Standard deviation	Standard error	95% CI	
Essay 1:					
Group 1: Torch casting - TC (n = 10)					
Weight (gr)	0.520	0.013	0.004	0.510	0.529
Failure diameter (cm)	0.155	0.013	0.004	0.145	0.165
Failure area (cm^2^)	0.019	0.003	0.001	0.016	0.021
Maximum load (KN)	0.290	0.057	0.018	0.249	0.331
Maximum load (kg)	30.090	6.097	1.928	25.728	34.451
Resistance obtained (Mpa)	157.207	30.363	9.601	135.486	178.928
Group 3: Electric arc casting - EAC (n = 10)					
Weight (gr)	0.558	0.017	0.005	0.545	0.570
Failure diameter (cm)	0.150	0.01	0.003	0.143	0.157
Failure area (cm^2^)	0.017	0.002	0.001	0.016	0.019
Maximum load (KN)	0.350	0.084	0.027	0.289	0.411
Maximum load (kg)	36.770	7.622	2.410	31.318	42.222
Resistance obtained (Mpa)	206.102	48.615	15.373	171.325	240.879
Mean difference of compressive strength between torch casting and electric arc casting group					
Difference EAC-TC	−48.895				
t	−2.698				
Degrees of freedom	18				
p value	0.015				
Essay 2					
Group 2: Torch casting -TC (n = 10)					
Weight (gr)	0.534	0.020	0.006	0.520	0.548
Failure diameter (cm)	0.166	0.016	0.005	0.155	0.178
Failure area (cm^2^)	0.022	0.004	0.001	0.019	0.025
Maximum load (KN)	0.290	0.074	0.023	0.237	0.343
Maximum load (kg)	29.020	4.407	1.394	25.867	32.173
Resistance obtained (Mpa)	136.303	43.349	13.708	105.293	167.313
Group 4: Electric arc casting - EAC (n = 10)					
Weight (gr)	0.550	0.034	0.011	0.525	0.575
Failure diameter (cm)	0.157	0.010	0.003	0.150	0.164
Failure area (cm^2^)	0.019	0.002	0.001	0.018	0.021
Maximum load (KN)	0.330	0.048	0.015	0.295	0.364
Maximum load (kg)	33.600	5.697	1.801	29.914	38.065
Resistance obtained (Mpa)	172.625	28.376	8.973	152.327	192.924
Mean difference of compressive strength between torch casting and electric arc casting group					
Difference EAC-TC	−36.322				
t	−2.217				
Degrees of freedom	18				
p value	0.040				

In Essay 2, posts fabricated using torch casting (Group 2) had an average weight of 0.534 grams, a failure diameter of 0.166 cm, and a failure area of 0.022 cm². The maximum load supported was 0.290 KN (29.020 kg), and the mean resistance obtained was 136.303 MPa. In contrast, posts from Group 4, fabricated using torch melting, had an average weight of 0.550 grams, a failure diameter of 0.157 cm, and a failure area of 0.019 cm². These posts supported a maximum load of 0.330 KN (33.600 kg) and had a mean resistance of 172.625 MPa. The average difference in compressive strength between the TC and EAC groups in this essay was -36.322 MPa, with a t-value of -2.217 and a p-value of 0.040, also indicating a significant difference.

Overall, the boxplots (Fig 1) illustrate that the electric arc casting technique consistently results in posts with higher compressive strength compared to those produced by torch casting. This difference is particularly significant in both essays.

### 3.2. Morphological, microstructural, and chemical characterization

**Fig 2:**
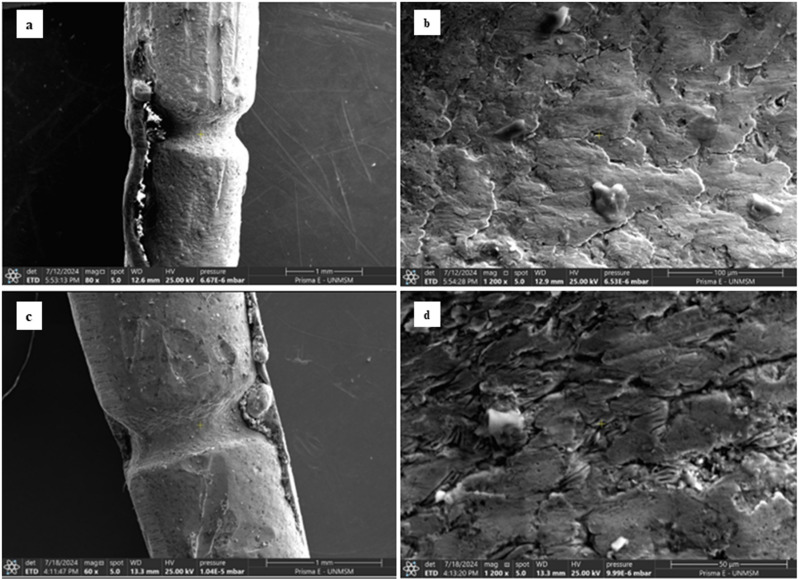
Comparative SEM of the selected areas from the electric arc and torch samples: a) Low magnification micrograph (80x) of the Electric Arc sample; b) High magnification micrograph (1200x) of the Electric Arc sample; c) Low magnification micrograph (60x) of the Torch sample; d) High magnification micrograph (1200x) of the Torch sample.

The morphological and structural analysis of the samples obtained by electric arc and torch reveals significant microstructural and compositional characteristics. Through scanning electron microscopy (SEM), it was observed that both samples exhibit a porous surface with an irregular topography (Fig 2). In the case of the sample obtained by electric arc, crystalline growths were identified within the copper matrix, suggesting a non-completely homogeneous stoichiometry. Similarly, in the sample obtained by torch, aluminum-enriched zones were detected within the copper matrix, also indicating a non-uniform distribution of elements.

**Fig 3:**
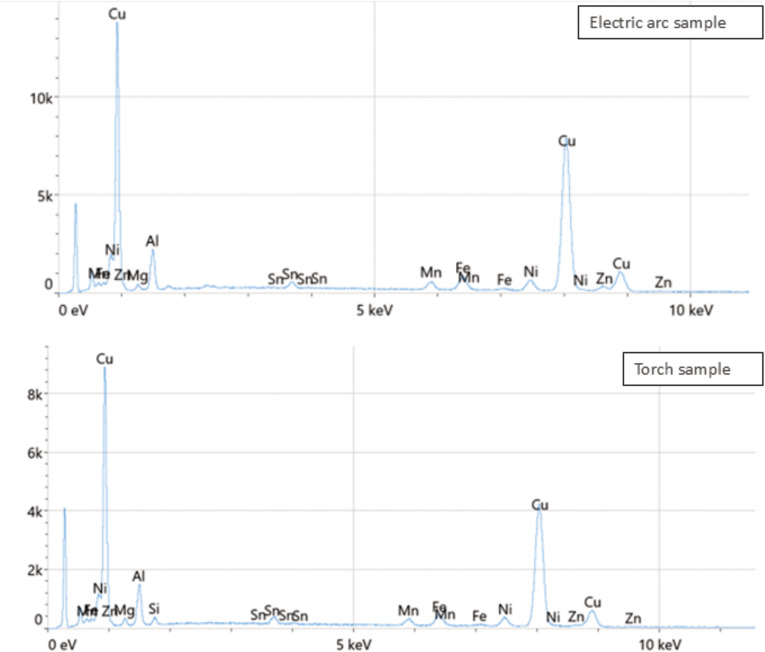
Comparative EDS spectrum analysis of the selected areas from the electric arc and torch samples.

Regarding chemical composition, analysis by energy-dispersive spectroscopy (EDS) showed that copper (Cu) is the predominant element in both samples (Fig 3). In the electric arc sample, copper accounts for 74.7% by weight, followed by aluminum (Al) at 13.1%, nickel (Ni) at 4.0%, and iron (Fe) at 3.3%. On the other hand, in the torch sample, copper is also the majority element at 76.2% by weight, followed by aluminum (10.0%), nickel (3.9%), and iron (3.2%). Both analyses confirm the presence of other elements in smaller proportions, such as magnesium (Mg), zinc (Zn), and silicon (Si).

**Fig 4:**
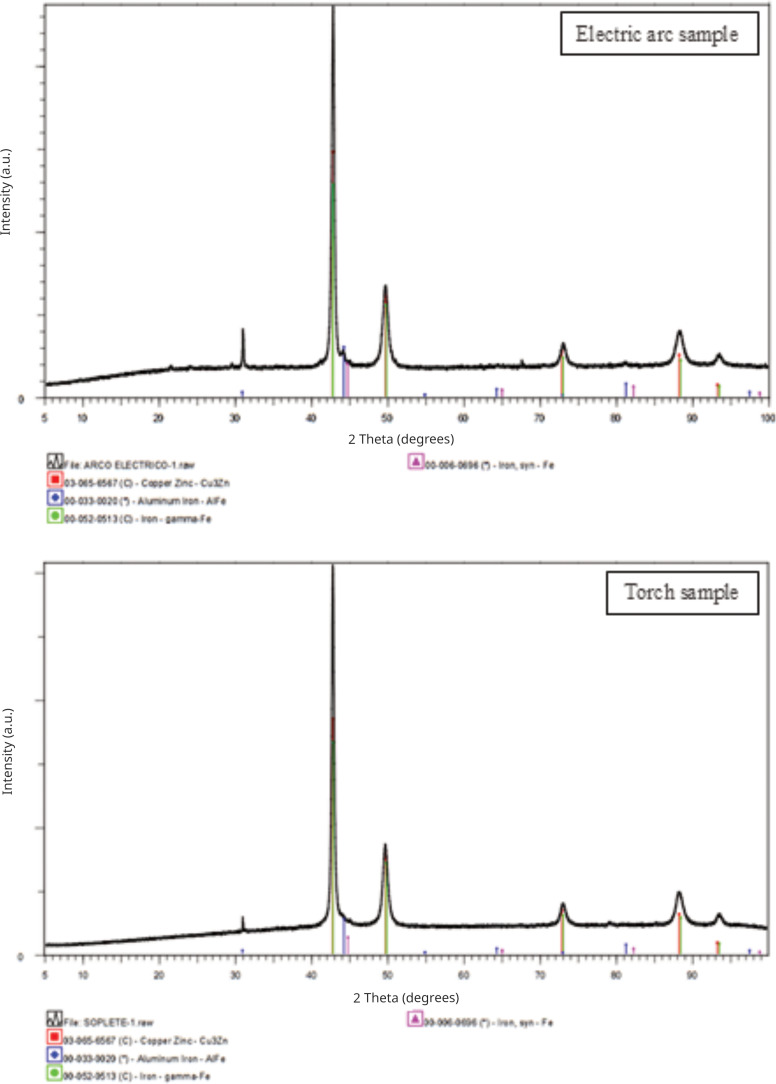
Comparative XRD diffractogram analysis of the selected areas from the electric arc and torch samples.

Phase analysis using X-ray diffraction (XRD) allowed the identification of the main crystalline phases present in the samples (Fig 4). In the electric arc sample, the predominant phases are Cu₃Zn (77.99%), gamma-Fe (9.06%), and AlFe (2.22%). In the torch sample, the main phases are Cu₃Zn (76.14%), gamma-Fe (23.20%), and AlFe (0.52%). These phases are supported by the corresponding JCPDS-PDF files. https://zoom.us/j/92272907672

Additionally, the crystallite size was calculated using the Scherrer equation, yielding very similar values for both samples. For the Cu₃Zn phase, the crystallite size was 14.9 nm in the electric arc sample and 14.88 nm in the torch sample. For the AlFe and gamma-Fe phases, the sizes were slightly larger in the electric arc sample (14.98 nm and 15.3 nm, respectively) compared to the torch sample (14.95 nm and 15.27 nm, respectively). These differences, although minimal, are consistent and suggest that the manufacturing method slightly influences the final microstructure of the material.

### 3.3. Cost analysis of casting techniques

The total cost per unit for torch casting is $1.465, while for electric arc casting it is $2.181. This indicates that the electric arc casting technique is more expensive, with a difference of $0.717 per unit compared to torch casting, primarily due to higher electricity consumption (Table 2).

**Table 2:** Cost analysis of casting techniques.

Casting technique	Fixed cost (per unit)		Variable cost (per unit)		Total cost (per unit)	Cost difference (per unit)
	Human resources	Depreciation (furniture, equipment, instruments e infrastructure)	Materials or supplies	Services		
Torch casting	$0.752	$0.015	$0.459	$0.237	$1.465	–
Electric arc casting	$0.752	$0.014	$0.005	$1.409	$2.181	$0.717

**Fig 5:**
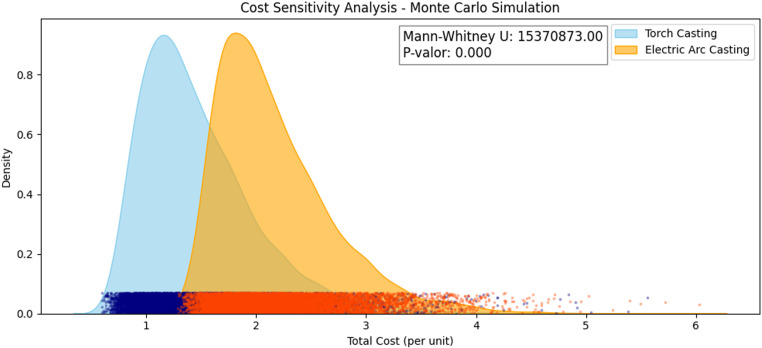
Cost sensitivity analysis – Monte Carlo simulation.

The cost sensitivity analysis using Monte Carlo simulation (Fig 5) compares Torch Casting and Electric Arc Casting. Adjusted distributions were used: Gamma (human resources, services), Log-Normal (materials), and PERT (depreciation, services). The Mann-Whitney U test reveals significant differences (p-value < 0.001), indicating Electric Arc Casting has higher and more variable costs than Torch Casting. This suggests that method selection impacts economic feasibility. The distributions used allow for modeling realistic cost uncertainty, reflecting variability and the occurrence of extreme events, which is essential for making informed decisions in manufacturing and cost management.

## 4. Discussion

The central result of this study is that posts fabricated using the electric arc casting (EAC) technique exhibit significantly higher compressive strength compared to those fabricated using torch casting (TC). In Essay 1, EAC posts supported a maximum load of 0.350 KN and showed a mean resistance of 206.102 MPa, compared to 0.290 KN and 157.207 MPa for TC posts. In Essay 2, EAC posts again outperformed TC posts with a mean resistance of 172.625 MPa versus 136.303 MPa. These findings indicate that the EAC technique produces stronger posts, which is crucial for the durability and efficiency of dental restorations.

In other main results, it was observed that the weight difference between posts fabricated by both techniques was minimal; however, EAC posts had slightly smaller failure diameters and areas in both essays. This suggests that the EAC technique not only improves compressive strength but also produces posts with more consistent physical characteristics. The smaller failure area could imply that EAC posts better distribute the load, which could explain their higher resistance capacity.

The morphological and microstructural analysis, performed using scanning electron microscopy (SEM), revealed a porous surface with irregular topography in both techniques. However, EAC samples displayed crystalline growths within the copper matrix, indicating a non-homogeneous stoichiometry, while TC samples showed aluminum-enriched zones, suggesting a non-uniform elemental distribution. These microstructural differences may explain the variations in mechanical performance between the two techniques.

Chemical composition analysis via energy-dispersive spectroscopy (EDS) identified copper (Cu) as the predominant element in both samples, with trace elements such as aluminum (Al), nickel (Ni), and iron (Fe) also present. X-ray diffraction (XRD) analysis further revealed distinct crystalline phases in each technique, with EAC samples exhibiting a higher proportion of Cu₃Zn and gamma-Fe phases compared to TC samples. These findings suggest that the EAC technique results in a more refined microstructure, which may contribute to its superior mechanical properties [20].

In the study by Ko KH [21] the effects of heat treatment on a Co-Cr alloy fabricated by selective laser melting (SLM) were investigated. Although their approach differs from ours in terms of fabrication technique, we share the importance of understanding how heat treatments affect the properties of dental materials. Al-Saleh S. et al. [22] compared metal frameworks fabricated with different techniques and evaluated their marginal fit. Although their approach differs from ours in terms of the fabrication technique, both studies highlight the relevance of precision in dental restorations. The study by Ahmadi E [23] focused on comparing PFM crowns fabricated with CAD/CAM techniques and the lost-wax technique. Although his focus is on marginal adaptation of crowns, his research shares the importance of evaluating different fabrication techniques in dentistry. Gonzales E. [24] investigated the flexural strength of NPG cast posts and glass posts in an in vitro study. Although he focused on posts rather than dental models, his study highlights the relevance of mechanical properties in dentistry, which we also explored in our work. In the study by Gaidwak BS et al. [25], cups fabricated by laser sintering of metal and lost wax were compared in terms of marginal fit and axial wall adaptability. Although their approach differs from our study in terms of materials and application, both highlight the importance of fit in dental restorations. The study by Yan X et al. [26] investigated the effects of heat treatments on Co-Cr metal-ceramic SLM alloys for dental prostheses. Although their focus is on the mechanical properties of the materials, they share with us the attention on how heat treatments affect these properties. Sarna-Bros K. et al. [11] compared the electroforming technique with traditional casting in dental prostheses. Although their approach differs from ours in terms of fabrication technique, both studies highlight the importance of marginal adaptation and fracture toughness in dental restorations. Iiyama K. [27] investigated the effects of casting conditions on the mechanical properties of a specific material. Although their focus is on a different material, they share with us the relevance of casting conditions on mechanical properties. Bizar i Ramoneda [28] carried out a study involving a detailed description of various alloy properties. Although their approach is broader than ours, both studies highlight the importance of understanding the properties of materials used in dentistry. In summary, these studies, conducted by researchers from different countries and at different times, provide a broader understanding of the field of dentistry and dental materials. Our study adds to this knowledge base by specifically exploring the compressive strength of cast dental models cast by electric arc and torch, thus contributing to the knowledge in this specific field [29].

We wanted to incorporate a cost analysis as well. The total cost per unit for torch casting is $1.467, while for electric arc casting it is $2.181. This indicates that the electric arc casting technique is more expensive, with a difference of $0.717 per unit compared to torch casting, primarily due to higher electricity consumption. The analysis of total costs per unit for torch casting and electric arc casting reveals a significant difference in expenses, primarily influenced by electricity consumption. This disparity highlights the higher cost associated with electric arc casting. The primary factor contributing to the increased cost of electric arc casting is its substantial electricity consumption. While both techniques incur costs related to human resources, depreciation, materials, and supplies, the variable cost associated with electricity in electric arc casting is notably higher. This suggests that, although electric arc casting may offer certain advantages in terms of precision or quality, it does so at a higher operational expense. For industries where cost-efficiency is paramount, torch casting may be the preferred method due to its lower overall cost [30]. However, in scenarios where the benefits of electric arc casting, such as potentially improved material properties or faster processing times, outweigh the additional cost, it might still be the more viable option. Decision-makers must therefore weigh the economic impact against the technical advantages to determine the most suitable casting technique for their specific needs [31].

Despite exhaustive efforts to control for multiple factors that could influence compressive strength, it is important to recognize that there are always variables not evaluated that could have inadvertently influenced the results. These uncontrolled variables may include individual variations in internal dental morphology, the complete clinical context (which includes factors such as adaptation to the root canal, dentin thickness, cementation, masticatory forces, and constant low-intensity loads), or even differences in the quality of materials used. It is critical to be aware of these possible unidentified influences and consider them when interpreting study results. These limitations may affect the generalizability of the results but do not completely invalidate them, as controlled tests were conducted, and robust statistical methods were used for analysis. The strengths of the study include a direct and systematic comparison of two different casting techniques, as well as the use of precise and reproducible measures to evaluate compressive strength [32].

A fundamental strength of this study lies in its contribution to the field of dentistry. By demonstrating that electric arc casting is associated with significantly higher compressive strength compared to torch casting, this study provides valuable evidence for dental professionals, particularly in low- to middle-income countries. This information can influence clinical practices and promote the adoption of alternative casting techniques that improve the quality and durability of cast dental models, which in turn could benefit patients and improve standards of dental care [33].

In conclusion, the results of this study indicate that the electric arc casting technique produces dental posts with significantly higher compressive strength compared to the torch casting technique. This suggests that the EAC technique could be preferred in clinical practice to improve the durability and efficiency of dental restorations.

As future research is considered, it is recommended also that the clinical implications of these findings be further explored, and consideration be given to expanding the study. Although corrosion resistance was not the focus of this study, it is important to highlight its relevance in dental applications, where materials are exposed to oral conditions that can induce corrosion. Corrosion resistance is a critical factor in ensuring the durability and biocompatibility of dental materials. Therefore, we suggest that future work includes corrosion resistance tests, as well as microbiological tests to evaluate the behaviour of materials in the presence of microorganisms found in the oral cavity. These additional tests would provide a more comprehensive understanding of the properties of the materials and their suitability for long-term clinical applications. This will allow for a more complete understanding of best practices in the fabrication of cast dental models and their impact on dental practice.

## 5. Conclusion

The study concludes that dental models cast using the electric arc technique exhibit superior compressive strength and refined microstructure compared to torch casting, enhancing restoration durability. However, electric arc casting is costlier due to higher electricity consumption. This research highlights the superior compressive strength and refined microstructure of dental models cast using the electric arc technique compared to torch casting, enhancing restoration durability, while acknowledging its higher operational costs.

## Supporting information

S1 File Cost structure for producing one unit of dental post by torch casting (TC) and electric arc casting (EAC). Cost structure fundition 22mar25.
(XLSX)

S2 File Database of physical and mechanical properties of dental posts fabricated by torch casting (TC) and electric arc casting (EAC). renamed_7ff19.
(DTA)
